# In Vitro and In Vivo Anti-Inflammatory Activities of *Tupistra chinensis* Baker Total Saponins

**DOI:** 10.3390/foods14172964

**Published:** 2025-08-25

**Authors:** Yajing Pu, Lin Li, Ru Wang, Qiuyi Qin, Jingwen Xu, Limin Xiang, Xiangjiu He, Yihai Wang

**Affiliations:** 1 School of Pharmacy, Guangdong Pharmaceutical University, Guangzhou 510006, Chinashuiyuntian0101@126.com (J.X.); 2 Guangdong Engineering Research Center for Lead Compounds & Drug Discovery, Guangzhou 510006, China; xianglimin88@163.com; 3 School of Chinese Materia Medica, Guangdong Pharmaceutical University, Guangzhou 510006, China

**Keywords:** *Tupistra chinensis* Baker, total saponins, extraction and enrichment, constituent, anti-inflammation

## Abstract

*Tupistra chinensis* Baker, traditionally used in southern China as a folk remedy for sore throat and pharyngitis, has long been consumed as a medicinal tea. Steroidal saponins are considered the major bioactive constituents of its rhizome, yet systematic studies on their extraction and biological effects remain scarce. In this study, total steroidal saponins from *T. chinensis* (TCS) were prepared through optimized extraction and enrichment processes. Response surface methodology identified optimal conditions, and subsequent purification with D-101 resin increased the saponin content to 67.3%. The anti-inflammatory activity of TCS was evaluated in vitro and in vivo. In LPS-stimulated RAW264.7 macrophages, TCS significantly inhibited nitric oxide production and downregulated the expression of iNOS, COX-2, and pro-inflammatory cytokines, partly through suppression of NF-κB activation. In a rat model of acute pharyngitis, TCS alleviated pathological symptoms, preserved mucosal integrity, and reduced NF-κB expression. These findings demonstrate that TCS possesses strong anti-inflammatory activity and may serve as a promising candidate for the development of functional foods or natural therapeutics aimed at pharyngitis management.

## 1. Introduction

*Tupistra chinensis* Baker, commonly known as Kaikoujian or Zhugenqi, is a perennial herb of the family Liliaceae mainly distributed in southern China, particularly in the Hubei Shennongjia region [1]. Its rhizome has been consumed for centuries in folk practice, often prepared as a tea to relieve sore throat and pharyngitis. According to the Chinese Materia Medica Dictionary, the dried rhizome has also been traditionally prescribed for diphtheria, traumatic injuries, and snake bites [2], reflecting its dual role as both medicine and food.

Modern studies have reported that extracts of *T. chinensis* exhibit diverse biological activities, including antifungal [3], antitumor [4], anti-inflammatory [5], and antioxidant effects [6]. A polysaccharide isolated from this plant was further shown to protect against ulcerative colitis [7]. Phytochemical analyses revealed that *T. chinensis* contains abundant saponins, flavonoids, and polysaccharides [2,8,9], with steroidal saponins recognized as the predominant bioactive constituents and potential contributors to its anti-inflammatory properties [10,11]. Our previous work identified several saponin monomers from *T. chinensis* rhizomes and confirmed their inhibitory effects on nitric oxide (NO) production in LPS-stimulated RAW264.7 cells [12,13,14,15].

Nevertheless, systematic investigations on the extraction, enrichment, chemical characterization, and bioactivity of total saponins from *T. chinensis* (TCS) remain limited. In particular, the anti-inflammatory effects of TCS have not been fully explored in both cellular and animal models, and their potential application as bioactive ingredients in functional foods has not been addressed. These knowledge gaps restrict the further development and utilization of *T. chinensis* as a nutraceutical resource.

Therefore, the present study aims to (i) optimize the extraction and enrichment process of TCS, (ii) characterize its major chemical constituents, and (iii) evaluate its anti-inflammatory activity in vitro and in vivo. This work provides a comprehensive scientific basis for promoting *T. chinensis* as a promising candidate for nutraceutical and functional food applications.

## 2. Materials and Methods

### 2.1. Reagents

Acetonitrile (chromatographic grade) was purchased from Oceanpak Chemical Co., Ltd. (Gothenburg, Sweden). *p*-Anisaldehyde and paraformaldehyde were acquired from Macklin Biochemical Co., Ltd. (Shanghai, China). Hematoxylin-eosin solution was obtained from Beyotime Biotechnology (Shanghai, China). Qingkailing granules were ordered from Guangzhou Baiyunshan Pharmaceutical Holding Co., Ltd. (Guangzhou, China). Other reagents (ethanol, ethyl acetate, sulfuric acid, 15% ammonia water) were of analytical grade or higher. All reagents for the MTT assay, Griess assay, qRT-PCR assay, Western blot assay, and immunofluorescence assay were the same as those used in our previous laboratory study [16].

### 2.2. Cells and Animals

RAW 264.7 cell line was purchased from Procell Life Science & Technology Co., Ltd. (Wuhan, China), with the catalog number CL-0190. The cell line was originally derived from ATCC: TIB-71 (ECACC: 91062702) and incubated in the same way as in our previous study [16].

Seventy female Wistar rats (180–220 g) were purchased from the Guangdong Experimental Animal Center. These animals had free access to food and distilled water for a week in a clean environment under a 12:12 h light–dark cycle with a temperature of 25 °C and relative humidity of 50% to 60%. All animal experiments were strictly approved by the Animal Ethics Committee of Guangdong Pharmaceutical University (No. SPF2017678).

### 2.3. Samples and Processing

The rhizomes of *T. chinensis* Baker were purchased from Shennongjia Forest Region (Wuhan, China) and authenticated by Professor Xiangjiu He of Guangdong Pharmaceutical University. The specimen (No. GDPU-NPR-2013002) has been deposited in the College of Pharmacy of Guangdong Pharmaceutical University (Guangzhou, China). After being dried, ground into powder, and passed through a 20-mesh sieve, the sample was stored in a cool, dry place for the following experiments.

### 2.4. TCS Extraction Process Optimization

#### 2.4.1. Single-Factor Experiments Design

Five factors and five levels were designed to investigate the extraction process of TCS from *T. chinensis* Baker rhizomes. Powder (0.5 g) was extracted using an ethanol reflux process under controlled conditions. Then, the extract was filtered and combined. The TCS yield was determined by UV–Vis analysis.

The factors and levels were as follows: ethanol concentration (55, 65, 75, 85, 95%), extraction time (30, 60, 90, 120, 150 min), liquid–solid ratio (5, 10, 15, 20, 25 mL/g), extraction times (1, 2, 3, 4, 5), and extraction temperature (50, 60, 70, 80, 90 °C) (Table 1).

#### 2.4.2. RSM Experimental Design

**Figure 1:**
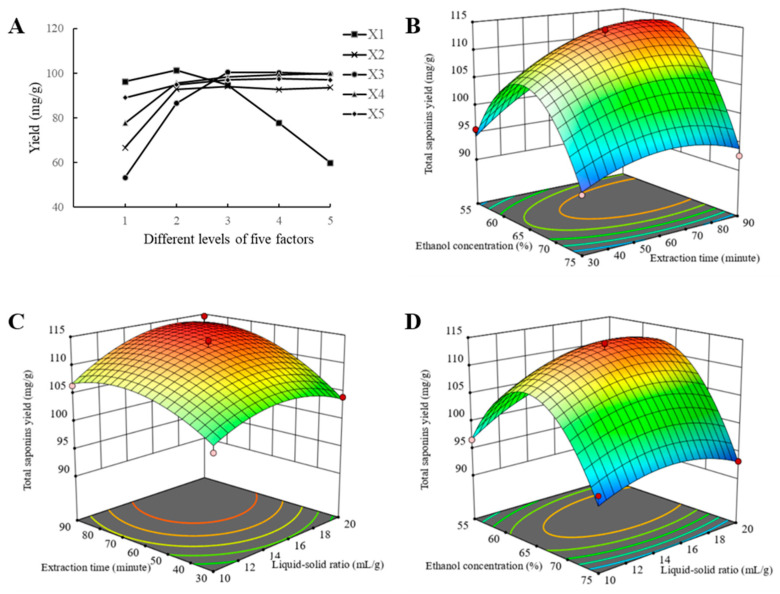
(**A**) The results of single-factor experiments (*n* = 3). X_1_: ethanol concentration (%), X_2_: extraction time (min), X_3_: liquid–solid ratio (mL/g), X_4_: extraction times and X_5_: temperature (°C). (**B**–**D**) 3D response surface diagram of response surface methodology (RSM).

Based on the results of the single-factor experiments shown in Figure 1A, a Box–Behnken design (BBD) with three variables and three levels was employed for further optimization [17,18]. The BBD comprised of twelve factorial points and five central points (Table 2).

### 2.5. TCS Enrichment Process Investigation

To improve the content of TCS, the crude extracts of *T. chinensis* Baker obtained under optimal conditions were enriched using macroporous resins [19]. The extraction solution was then evaporated to dryness and redissolved in water.

#### 2.5.1. Static Adsorption and Desorption Design

The static adsorption and desorption capacities of five types of microporous resins with different polarities, including D-101, AB-8, LX-68M, DM-130, and HPD-500, were investigated to select the most suitable resins [20]. A total of 5 g of pretreated resins and 30 mL of crude saponin solution (equal to 10.8 mg/mL saponins) were placed into a conical flask with a stopper. Then, the flasks were shaken in a thermostatic oscillator at 120 r/min and 30 °C for 24 h. After achieving adsorption equilibrium, the resins were filtered and washed with DDW twice and then desorbed with 50 mL of 90% ethanol for 24 h. The content of saponins in the filtrate was determined, and the adsorption capacity (*Q*), adsorption rate (*E*_1_), and desorption rate (*E*_2_) were calculated according to the following equations, respectively:

$$Q(mg/g)=\frac{(C_{0}-C_{a})\times V}{M}$$

$$E_{1}(\%)=\frac{C_{0}-C_{a}}{C_{0}}\times 100$$

$$E_{2}(\%)=\frac{C_{d}\times V_{d}}{{(C}_{0}-C_{a})\times V}\times 100$$

where *C*_0_ and *C_a_* represent the saponin concentration in the initial and equilibrium solution (mg/mL), *V* is the volume of the initial solution (mL), *M* is the mass of the resins (g), and *C_d_* and *V_d_* are the saponin concentration and volume of the desorption solution, respectively. Recovery was quantified using the following equation:

$$R(\%)=\frac{C_{d}\times V_{d}}{C_{0}\times V}\times 100$$

#### 2.5.2. Enrichment of TCS on Macroporous Resins

Enrichment experiments were conducted on glass columns packed with the selected resins to optimize the purification conditions of crude saponins. Different initial concentrations of crude saponins (2.7, 5.4, 8.1, 10.8, and 13.5 mg/mL) were dynamically absorbed and eluted with 70% ethanol to evaluate recovery efficiency. The influence of the resin column diameter–height ratio (1:2.5, 1:5, 1:7.5, 1:10, and 1:12.5) was also examined using 2BV crude saponins (10.8 mg/mL), followed by elution with 5BV 70% ethanol. In addition, different ethanol concentrations (10%, 30%, 50%, 70%, and 95%) were applied successively to determine the optimal elution solvent. Finally, the effect of eluent volume was investigated by eluting with 5BV 70% ethanol and collecting each 1BV fraction for analysis.

### 2.6. Total Saponin Content Determination

The content of total saponins in TCS from *T. chinensis* Baker was quantified by using the *p*-anisaldehyde–sulfuric acid method [21]. The sample solution (0.3 mL) was dried in a water bath and dissolved in 2 mL of ethyl acetate. Then, 1 mL of reagent A (0.5 mL of *p*-anisaldehyde plus 99.5 mL of ethyl acetate) and 1 mL of reagent B (50 mL of concentrated sulfuric acid plus 50 mL of ethyl acetate) were added. After the solution was fully shaken, the test tubes were placed in a water bath at 60 °C for 15 min and then cooled in an ice bath. The absorbance was measured at 430 nm using a 2550 UV–Vis spectrophotometer (Shimadzu Co., Ltd., Kyoto, Japan). To determine the content of TCS as accurately as possible, Compound **2**, one of the most abundant saponins in *T. chinensis* Baker, was selected as the reference substance. Finally, the extraction yield and content of TCS (*C_s_*) were calculated according to the following equations:

$$Yield(mg/g)=\frac{C\times V_{e}}{m}$$

$$C_{s}(\%,w/w)=\frac{m_{s}}{m_{e}}\times 100$$

where *C* is the concentration of saponins in the extracting solution (mg/mL), *V_e_* is the volume of the solution (mL), *m* is the mass of *T. chinensis* Baker powder (g), *m_s_* represents the weight of saponins in the product, and *m_e_* is the weight of the product.

### 2.7. Chemical Constituent Analysis of TCS

#### 2.7.1. Sample Preparation

TCS samples acquired under the optimal preparation conditions were dissolved in 17% acetonitrile. After filtration and volume adjustment, the mass concentration of the TCS sample was 10.0 mg/mL.

#### 2.7.2. HPLC Conditions and Parameters

Identification and quantification of saponins in TCS were performed on the Shimadzu HPLC system coupled with a Sedex-75 evaporative light-scattering detector (Dikema Technology Co., Ltd., Beijing, China). A COSMOSIL 5C_18_-MS-II analysis column (4.6 ID × 250 mm) was used for chromatographic separation [22]. The mobile phase was composed of acetonitrile (A) and water (B), and the gradient program was as follows: 0–10 min, 17~19% A; 10–30 min, 19~23% A; 30–40 min, 23~27% A; 40–45 min, 27~40% A; 45–55 min, 40~100% A; 55–60 min, 100% A. The temperature of the column was set to 25 °C, and the flow rate was 1.0 mL/min. The drift tube temperature of ELSD was set at 95 °C, and the gas pressure was 1.5 bar. Nine representative saponins of *T. chinensis* Baker were selected for quantitative analysis. These compounds were chosen due to their relatively high abundance in the extract and their relevance as major characteristic constituents, as documented in previous phytochemical studies [12,13,14].

### 2.8. In Vitro Evaluation of Anti-Inflammatory Activity 

TCS was dissolved into different concentrations by DMSO, and its effect on LPS-induced RAW264.7 cells was investigated through MTT assay, Griess assay, qRT-PCR assay, Western blot assay, and immunofluorescence assay. Detailed procedures followed the methods reported by our laboratory study [16].

### 2.9. In Vivo Evaluation of Anti-Inflammatory Activity 

#### 2.9.1. Acute Pharyngitis Model Establishment and Pharmacological Intervention

Seventy rats were randomly divided into seven groups, including a blank control group (BC), two model groups (Model 1 and Model 2, AP), a Qingkailing group (QKL), and three TCS treatment groups with different doses (high, medium, and low: TCS-H, TCS-M, and TCS-L). The acute pharyngitis model was induced by spraying 15% ammonia water on the pharynx of rats twice per day for three consecutive days [23], while rats in the blank control group were sprayed with the same amount of normal saline. On the fourth day, rats in Model 1 were sacrificed, and pharyngeal tissues were collected for subsequent analysis. The treatment groups were orally administered Qingkailing (2 g/kg) or TCS at high (140 mg/kg), medium (70 mg/kg), and low (35 mg/kg) doses for five consecutive days. The TCS doses were determined based on preliminary experiments and previous reports on the effective anti-inflammatory activity of saponin fractions, ensuring both efficacy and safety [24,25]. Meanwhile, the blank control and Model 2 groups received an equivalent volume of normal saline. Twenty-four hours after the last administration, all rats were treated in the same manner as in Model 1.

#### 2.9.2. Behavioral Study and Pharyngeal Tissue Pathological Evaluation

Based on the behavioral and physical marking criteria (see Table 3), scores corresponding to changes in appearance indexes, including activity, mouth hair loss, salivary secretion, mouth scratch, and pharyngeal swelling, were recorded [26].

#### 2.9.3. HE Staining of Pharyngeal Tissue

For histological evaluation, pharyngeal tissues were infused in 4% paraformaldehyde fixing solution for 24 h at room temperature and then dehydrated and embedded in paraffin. Embedded tissues were cut into 4 μm-thick slices using a Leica RM2235 rotary microtome (Wetzlar, Germany) and stained sequentially with hematoxylin-eosin staining [27]. Inflammation changes in pharyngeal tissues were observed using a microscope.

#### 2.9.4. Immunohistochemistry Staining of Pharyngeal Tissue

For immunohistochemistry evaluation, the formalin-fixed specimens were embedded in paraffin and cut into 4 μm-thick sections. These sections were dewaxed with xylene and rehydrated in a gradient series of alcohols. After being sealed with endogenous peroxidase sealing solution at room temperature for 10 min, the slices were immersed in antigen repair solution and heated to a gentle boil for about 10 min. After being blocked with 5% BSA for 30 min, the slices were incubated with the primary antibody at 4 °C overnight. The next day, the slices were incubated with the corresponding secondary antibody for 2 h, and then a 3,3′-diaminobenzidine (DAB) kit and hematoxylin were used for color reaction. Pharyngeal tissues were then observed under a microscope.

### 2.10. Statistical Analysis

Each experiment was repeated at least three times, and the results were expressed as mean ± SD. Comparisons between groups were calculated by SPSS 23 software. The differences were considered statistically significant when *p* < 0.05 and very significant when *p* < 0.01.

## 3. Results and Discussions

### 3.1. Single-Factor Experiments

To determine the appropriate parameter ranges, five single factors were investigated: ethanol concentration, extraction time, liquid–solid ratio, extraction frequency, and extraction temperature (Figure 1). The yield of TCS increased as ethanol concentration (X_1_) rose from 55% to 65% and then decreased at higher concentrations, likely due to the polarity match between saponins and 65% ethanol. Extraction time (X_2_) positively influenced yield from 30 to 90 min, with the increase slowing beyond 60 min. Similarly, the liquid–solid ratio (X_3_) enhanced yield progressively from 5 to 15 mL/g, reaching a maximum at 15 mL/g. For extraction frequency (X_4_) and extraction temperature (X_5_), the yield peaked at three extraction cycles and was relatively higher at 70 °C. Based on these results, ethanol concentration (55, 65, 75%), liquid–solid ratio (10, 15, 20 mL/g), and extraction time (60, 90, 120 min) were selected for subsequent RSM optimization.

### 3.2. RSM Optimization

#### 3.2.1. Model Fitting and Statistical Analysis

A second-order polynomial model was constructed to describe the extraction variables and TCS yield, based on regression analysis of the experimental data (Table 1) using Design Expert 12.0.3.0 [28]. The fitted equation was

$$Y=113.00-3.77\times X_{1}+1.98\times X_{2}+3.55\times X_{3}-1.90\times X_{1}X_{2}-2.65\times X_{1}X_{3}+1.35\times X_{2}X_{3}-13.00\times {X_{1}}^{2}-3.2\times {X_{2}}^{2}-3.25\times {X_{3}}^{2}.$$

The ANOVA results revealed that the model was highly significant (*p* < 0.01), with a non-significant lack of fit (*p* = 0.1706, Table 4), confirming the adequacy of the regression model. The coefficient of determination (*R*^2^ = 0.9845) indicated that only 1.55% of the variations was unexplained, while the adjusted *R*^2^ (0.9647) demonstrated strong consistency between predicted and experimental values. Among the tested variables, ethanol concentration (X_1_), liquid–solid ratio (X_2_), and extraction time (X_3_) were highly significant factors, with notable interactions observed between X_1_X_2_ and X_1_X_3_. These results confirmed that the constructed model was robust and reliable for optimizing the extraction process [29].

#### 3.2.2. Response Surface Analysis

Three-dimensional response surface plots (Figure 1B–D) illustrate the interactions among the extraction parameters. All plots were convex with a distinct maximum, confirming that the chosen variable ranges were appropriate. The optimal extraction conditions predicted were an ethanol concentration of 62.75%, a liquid–solid ratio of 15.03 mL/g, and an extraction time of 73.07 min. Extraction frequency (three times) and temperature (70 °C) were determined from the single-factor experiments. The predicted maximum yield was 114.7 mg/g·ss.

#### 3.2.3. Validation of Optimal Conditions

To validate the model, three replicate experiments were performed under slightly adjusted conditions (ethanol concentration 63%, liquid–solid ratio 15 mL/g, extraction time 75 min). The experimental yield was 113.7 ± 0.9 mg/g, closely matching the predicted value. These results confirmed the accuracy of the model. The crude extract contained 18.1% saponins.

### 3.3. Enrichment Process

#### 3.3.1. Results of Static Adsorption and Desorption Tests

The static adsorption and desorption capacities of five microporous resins were assessed (Table 5). All resins demonstrated high adsorption capacity, but the D-101 resin showed the best balance of adsorption and desorption efficiency. Thus, D-101 was selected for enrichment.

#### 3.3.2. Enrichment of TCS

**Figure 2:**
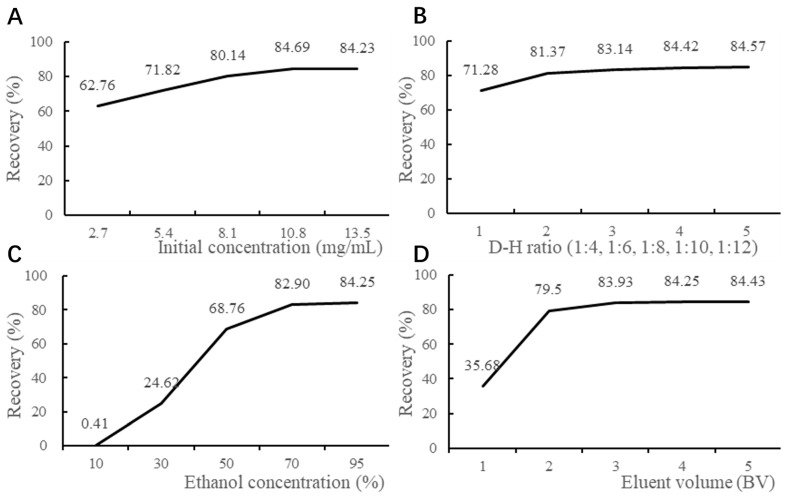
The results of enrichment conditions investigation (*n* = 3). (**A**) Effect of initial concentration on recovery; (**B**) Effect of D–H ratio on recovery; (**C**) Effect of ethanol concentration on recovery; (**D**) Effect of eluent volume on recovery.

Saponin recovery was measured using the *p*-anisaldehyde–sulfuric acid method with UV–Vis spectrophotometry. As shown in Figure 2A, recovery increased with initial saponin concentration up to 10.8 mg/mL, after which it declined, likely due to saturation. Therefore, 10.8 mg/mL was chosen. The diameter-to-height ratio had little effect (Figure 2B), but a 1:10 ratio was selected for practicality.

Elution studies (Figure 2C) showed that 10% ethanol failed to elute saponins, while ethanol concentrations above 70% caused excessive elution of non-saponin impurities. Hence, 10% ethanol was used to wash impurities, and 70% ethanol was used for saponin elution. Increasing the elution volume of 70% ethanol improved recovery, but gains plateaued beyond three bed volumes (BV) (Figure 2D). Therefore, 3 BV was selected.

The eluent was dried to yield a yellow powder, defined as TCS. After enrichment, the saponin content increased from 18.1% in the crude extract to 67.3% in TCS, with an overall recovery of 84.82%.

### 3.4. Chemical Constituent of TCS

**Figure 3:**
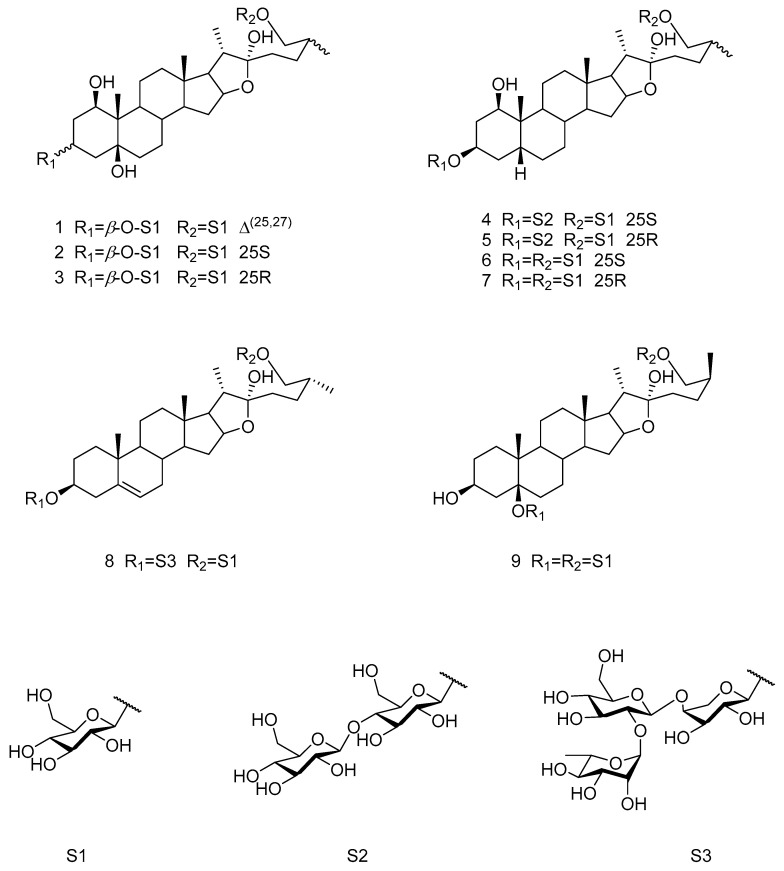
The chemical structures of compounds **1–9**.

**Figure 4:**
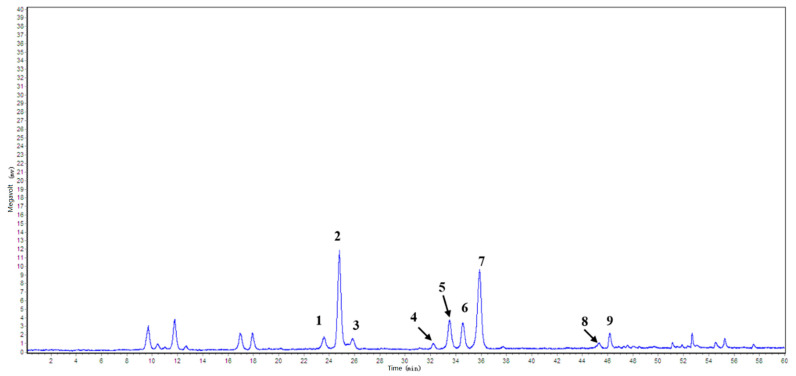
HPLC-ELSD chromatographic profile of TCS from *T. chinensis* Baker. Peaks **1**–**9** were identified based on their retention times using reference standards, and their corresponding constituents are listed in Table 6.

Saponins previously isolated from *T. chinensis* Baker in our laboratory were used as reference standards [12,13,14,15]. Their structures were verified using spectroscopic methods, and nine abundant and representative saponins were selected for quantification (Figure 3). These compounds were identified as the principal constituents of TCS (Figure 4), with their retention times and relative contents summarized in Table 6. Compounds **2** and **6** were the most abundant, each comprising ~16% of the total. Collectively, the nine saponins accounted for over 62% of the TCS extract.

### 3.5. In Vitro Anti-Inflammatory Effects of TCS 

**Figure 5:**
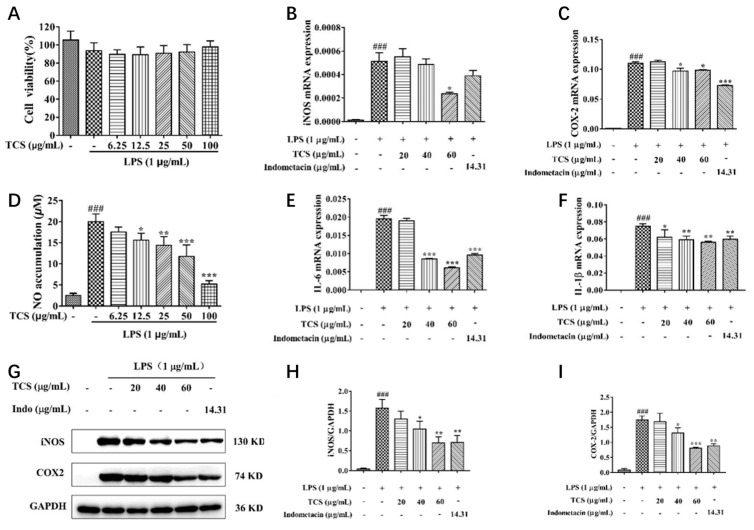
Effects of TCS on LPS-induced RAW264.7 cells. (**A**) Cell viability, (**D**) NO production, (**B**,**C**,**E**,**F**) cytokine expression, and (**G**–**I**) protein expression. Cells were treated with 20–40 μg/mL TCS and 1 μg/mL LPS for 24 h. Cell viability was assessed by MTT assay, NO production was determined using Griess reagent, and the mRNA and protein levels of iNOS, COX-2, IL-1*β*, and IL-6 were quantified using qRT-PCR and Western blotting. Data are expressed as mean ± SD (*n* = 3). ### *p* < 0.001 vs. control group; * *p* < 0.05, ** *p* < 0.01, *** *p* < 0.001 vs. LPS group.

To exclude potential cytotoxicity, the effect of TCS on the viability of RAW 264.7 cells was assessed using the MTT assay. As shown in Figure 5A, TCS at concentrations of 6.25–100 μg/mL did not significantly affect cell viability, indicating that the subsequent anti-inflammatory effects were not due to cytotoxicity.

Nitric oxide (NO), a key inflammatory mediator, was markedly increased after LPS stimulation. TCS treatment (12.5–100 μg/mL) significantly reduced NO accumulation in a dose-dependent manner, with approximately 50% inhibition at 50 μg/mL (Figure 5C). Consistently, TCS suppressed both the mRNA and protein expression of the upstream enzymes iNOS and COX-2 (Figure 5B,D–I). At higher concentrations (60 μg/mL), the inhibitory effect of TCS on protein expression was comparable to that of indomethacin.

Pro-inflammatory cytokines IL-1*β* and IL-6 were also elevated in LPS-stimulated cells. qRT-PCR analysis showed that TCS downregulated the expression of both cytokines in a dose-dependent manner (Figure 5E,F). Notably, the inhibitory effect of TCS on IL-1*β* at 40 μg/mL was stronger than that of indomethacin.

**Figure 6:**
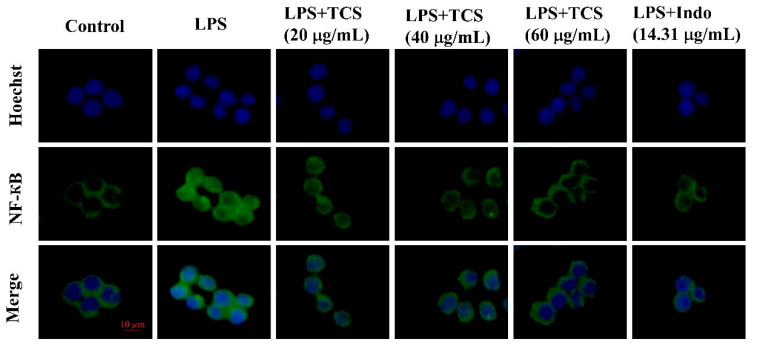
Effects of TCS on NF-κB in LPS-induced RAW264.7 cells. Cells were treated with 20–40 μg/mL TCS and 1 μg/mL LPS for 4 h. NF-κB translocation was detected by immunofluorescence staining. (Scale bar = 10 μm).

Given the central role of NF-κB in regulating inflammatory mediators, its activation was further examined. Immunofluorescence staining revealed pronounced NF-κB nuclear translocation upon LPS induction, whereas TCS treatment markedly suppressed this translocation (Figure 6), suggesting that TCS blocks NF-κB activation.

In summary, TCS significantly attenuated the LPS-induced inflammatory response in RAW 264.7 cells by inhibiting NF-κB nuclear translocation and downregulating its downstream targets, including iNOS, COX-2, IL-1*β*, and IL-6. These findings support the potential of TCS as a natural anti-inflammatory agent with efficacy comparable to that of indomethacin.

### 3.6. In Vivo Anti-Inflammatory Effects of TCS 

The animal model of acute pharyngitis was established according to the Specification for the Preparation of Animal Models of Acute Pharyngitis (Draft) proposed by the Experimental Pharmacology Committee of the Chinese Association of Traditional Chinese Medicine. This draft summarizes the clinical characteristics of acute pharyngitis in both traditional Chinese medicine and Western medicine, and outlines performance indices (fur loss in the mouth, salivation, poor spirit, throat swelling) and pathological indices (e.g., HE staining) as primary indicators. Each performance index can be graded as mild, moderate, or severe.

In a preliminary test, both male and female mice were induced with acute pharyngitis by exposure to 15% ammonia water without treatment. Marked sex differences were observed: female mice developed more severe and persistent symptoms lasting 7–10 days, whereas most male mice began to recover spontaneously from day 3. Based on these observations, female mice were selected for subsequent experiments.

#### 3.6.1. TCS Alleviated the Pathological Symptoms of Acute Pharyngitis in Rats

**Figure 7:**
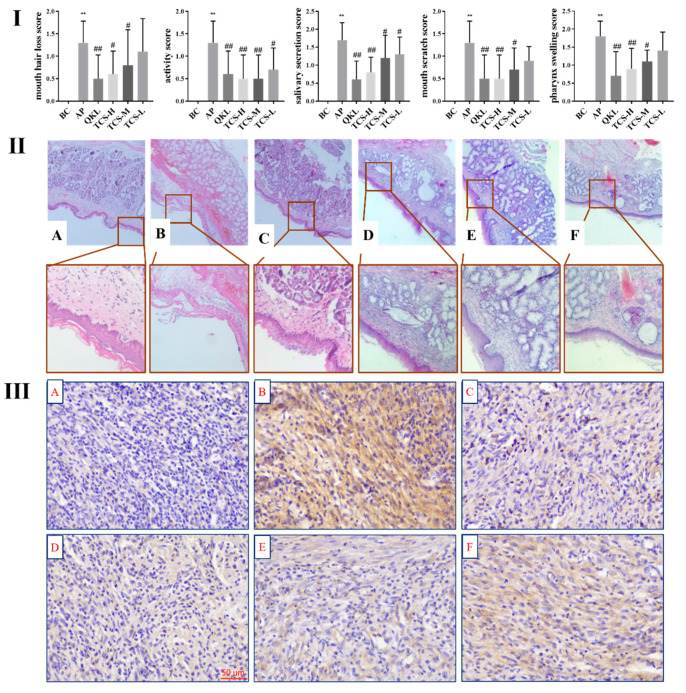
(**I**) Modulation of TCS on inflammatory symptoms of ammonia-induced acute pharyngitis in rats. Scores are expressed as mean ± SD, ** *p* < 0.01 versus the control group; ^##^ *p* < 0.01; ^#^ *p* < 0.05 versus the model group. (**II**) Effect of TCS on histopathological changes of ammonia-induced acute pharyngitis rats (*n* = 10). (**A**) BC group, (**B**) AP group, (**C**) Qingkailing group, (**D**) TCS-H group, (**E**) TCS-M group, (**F**) TCS-L group. The pharyngeal tissues were fixed, dehydrated, embedded, cut, and then stained with HE. The images were observed under a microscope at 50× and 200× magnification. (**III**) Effect of TCS on the expression of NF-κB in acute pharyngitis rats (*n* = 10). (**A**) BC group, (**B**) AP group, (**C**) Qingkailing group, (**D**) TCS-H group, (**E**) TCS-M group, (**F**) TCS-L group. The images were observed under a microscope at 20× magnification.

On the eighth day, the severity of pharyngitis was assessed according to behavioral and clinical criteria. Rats in the blank control group exhibited normal activity and intact pharyngeal mucosa [26]. After model induction, rats showed reduced weight and activity, increased salivation and mouth scratching, redness, swelling, and ulceration of the pharyngeal mucosa, and varying degrees of fur loss around the mouth. Treatment with TCS, particularly at higher doses, significantly alleviated these pathological symptoms, as reflected by reduced pathology scores (Figure 7I).

#### 3.6.2. TCS Relieved the Pathological Damage in Acute Pharyngitis Rats

Hematoxylin-eosin (HE) staining was performed to evaluate histopathological changes in pharyngeal tissues [23]. Compared with the blank control group, model rats exhibited massive inflammatory cell infiltration, atrophy of salivary glands, connective tissue proliferation, and occasional hemorrhage (Figure 7II). Treatment with TCS or Qingkailing granules markedly improved these histopathological alterations in a dose-dependent manner. Notably, high-dose TCS (140 mg/kg) produced improvements comparable to those of Qingkailing granules.

#### 3.6.3. TCS Reduced the Expression of NF-κB in Acute Pharyngitis Rats

NF-κB is a central transcription factor regulating inflammatory mediators and plays a pivotal role in the inflammatory response. Immunohistochemistry staining was applied to evaluate NF-κB expression in pharyngeal tissues. Compared with the blank control group, NF-κB expression was markedly increased after model induction, whereas treatment with TCS significantly reduced NF-κB expression (Figure 7III).

Although immunohistochemistry provides valuable visual evidence of protein expression changes, it is not a fully quantitative method, and no universally established approach exists for precisely assessing NF-κB activity in vivo. Therefore, our results suggest—but do not definitively prove—that the alleviation of pathological symptoms and tissue damage by TCS may be associated with modulation of the NF-κB pathway. Further mechanistic studies employing more specific molecular assays are warranted to confirm the pathway involvement.

Interestingly, the efficacy of high-dose TCS (140 mg/kg) in reducing NF-κB expression and improving pathological outcomes was comparable to that of Qingkailing granules, a traditional Chinese medicine widely used for treating sore throat.

## 4. Conclusions

In this study, we systematically investigated the extraction process, chemical composition, and anti-inflammatory effects of total saponins (TCS) derived from *Tupistra chinensis* rhizomes. Optimal extraction conditions were established—63% ethanol, 15 mL/g liquid–solid ratio, 75 min extraction time, three extractions at 70 °C—yielding 113.7 mg/g of TCS. Resin enrichment increased saponin content from 18.1% to 67.3%, with a recovery of 84.7%. HPLC–ELSD analysis identified nine major saponin constituents.

TCS demonstrated potent anti-inflammatory activity in vitro by suppressing inflammatory mediators in RAW264.7 cells and inhibiting NF-κB activation. In vivo, TCS effectively alleviated pharyngeal tissue pathology and preserved mucosal integrity in a rat model of acute pharyngitis.

Taken together, this work establishes a robust, reproducible preparation method and elucidates the key bioactive saponins in TCS. Importantly, it highlights TCS as a promising bioactive ingredient for designing functional foods or nutraceutical products targeting inflammation-related health benefits.

## Figures and Tables

**Table 1:** Factors and levels of Box–Behnken experiments for the optimization of extraction conditions.

Factors	−1	0	1
X_1_: Ethanol concentration (%)	55	65	75
X_2_: Liquid–solid ratio (mL/g)	10	15	20
X_3_: Extraction time (min)	30	60	90

**Table 2:** The experimental results for Box–Behnken design (*n* = 3).

Run	X_1_	X_2_	X_3_	Total Saponin Yield (mg/g)
1	65	10	30	101.2
2	65	15	60	114.0
3	75	15	30	91.6
4	55	15	90	107.2
5	75	10	60	94.6
6	55	20	60	102.8
7	65	15	60	114.2
8	55	10	60	96.6
9	75	20	60	93.2
10	55	15	30	95.6
11	75	15	90	92.6
12	65	10	90	106.4
13	65	15	60	111.2
14	65	15	60	112.8
15	65	20	30	104.0
16	65	15	60	112.8
17	65	20	90	114.6

**Table 3:** Behavioral and physical score criteria in ammonia-induced pharyngitis rat models.

Score	Mouth Hair Loss	Activity	Salivary Secretion	Mouth Scratch	Pharynx Swelling
0	Normal	Normal	Normal	Normal	Normal
1	Slight	Sligh	Sligh	Sligh	Sligh
2	Severe	Severe	Severe	Severe	Severe

**Table 4:** ANOVA of the regression model for the extraction yields of saponins.

Source	Sum of Squares	df	Mean Square	F-Value	*p*-Value
Model	2.86	9	0.3178	49.52	<0.0001
X_1_	0.2850	1	0.2850	44.41	0.0003
X_2_	0.0780	1	0.0780	12.16	0.0102
X_3_	0.2521	1	0.2521	39.27	0.0004
X_1_X_2_	0.0361	1	0.0361	5.62	0.0495
X_1_X_3_	0.0702	1	0.0702	10.94	0.0130
X_2_X_3_	0.0182	1	0.0182	2.84	0.1358
X_1_^2^	1.78	1	1.78	277.19	<0.0001
X_2_^2^	0.1078	1	0.1078	16.80	0.0046
X_3_^2^	0.1112	1	0.1112	17.32	0.0042
Residual	0.0449	7	0.0064		
Lack of Fit	0.0305	3	0.0102	2.83	0.1706
Pure Error	0.0144	4	0.0036		
Cor Total	2.91	16			
R^2^ = 0.9845, R^2^_Adj_ = 0.9647, C.V.% = 1.54					

**Table 5:** Static adsorption and desorption capacities of total saponins by different types of resins (*n* = 3).

Resin Type	Polarity	Adsorption Capacity/mg/g	Adsorption Rate/%	Desorption Rate/%	Recovery/%
D-101	Non	57.86 ± 0.42	89.29 ± 0.13	92.04 ± 1.97	82.13 ± 1.65
AB-8	Weak	55.54 ± 0.20	88.79 ± 0.06	76.83 ± 3.81	68.36 ± 3.24
LX-68M	Medium	58.82 ± 1.24	90.77 ± 0.38	77.68 ± 3.46	70.51 ± 2.77
DM-130	Medium	55.42 ± 0.18	85.51 ± 0.57	79.76 ± 2.51	68.21 ± 3.30
HPD-500	Strong	57.00 ± 0.36	87.95 ± 0.11	72.44 ± 3.85	72.44 ± 3.47

**Table 6:** Chemical constituents of TCS from *T. chinensis* Baker.

Compd.	Name	t_R_ (min)	Equation	Content (%)	RSD (%)
**1**	26-*O*-*β*-D-glucopyranosyl-furost-25(27)-en-1*β*,3*β*,5*β*,22*α*,26-pentaol-3-*O*-*β*-D-glucopyranoside	23.557	y=4131.7x−1716 (R^2^ = 0.9994)	4.82 ± 0.14	2.90
**2**	(25*S*)-26-*O*-*β*-D-glucopyranosyl-furost-1*β*,3*β*,5*β*,22*α*,26-pentaol-3-*O*-*β*-D-glucopyranoside	24.740	y=7997.1x−4006 (R^2^ = 0.9994)	16.08 ± 0.66	4.10
**3**	(25*R*)-26-*O*-*β*-D-glucopyranosyl-furost-1*β*,3*β*,5*β*,22*α*,26-pentaol-3-*O*-*β*-D-glucopyranoside	25.823	y=10835x−4946 (R^2^ = 0.9992)	2.87 ± 0.12	4.18
**4**	(5*β*,25*S*)-26-*O*-*β*-D-glucopyranosyl-furost-1*β*,3*β*,22*α*,26-tetraol-3-*O*-*β*-D-glucopyranosyl-(1→4)-*β*-D-glucopyranoside	32.207	y=8135x−5023 (R^2^ = 0.9991)	3.78 ± 0.08	2.07
**5**	(5*β*,25*R*)-26-*O*-*β*-D-glucopyranosyl-furost-1*β*,3*β*,22*α*,26-tetraol-3-*O*-*β*-D-glucopyranosyl-(1→4)-*β*-D-glucopyranoside	33.490	y=8018.6x−2382 (R^2^ = 0.9991)	5.88 ± 0.02	0.34
**6**	(5*β*,25*S*)-26-*O*-*β*-D-glucopyranosyl-furost-1*β*,3*β*,22*α*,26-tetraol-3-*O*-*β*-D-glucopyranoside	34.557	y=1753.3x−646.1 (R^2^ = 0.9991)	16.22 ± 0.48	2.96
**7**	(5*β*,25*R*)-26-*O*-*β*-D-glucopyranosyl-furost-1*β*,3*β*,22*α*,26-tetraol-3-*O*-*β*-D-glucopyranoside	35.867	y=16387x−7128 (R^2^ = 0.9997)	8.54 ± 0.27	3.16
**8**	(25*R*)-26-*O*-*β*-D-glucopyranosyl-22*α*-methoxyl-furost-5(6)-en-3*β*,26-diol-3-*O*-*α*-L-rhamnopyranosyl-(1→2)-*β*-D-glucopyranosyl-(1→4)-*β*-D-galactopyranoside	45.323	y=12957x−4852 (R^2^ = 0.9989)	2.34 ± 0.05	2.14
**9**	(25*S*)-26-*O*-*β*-D-glucopyranosyl-furost-3*β*,5*β*,22*α*,26-tetraol-5-*O*-*β*-D-glucopyranoside	46.223	y=13930x−4241 (R^2^ = 0.9997)	2.40 ± 0.04	1.67

## Data Availability

The original contributions presented in this study are included in the article. Further inquiries can be directed to the corresponding author(s).
